# The Effect of File Size and Type and Irrigation Solutions on the Accuracy of Electronic Apex Locators: An* In Vitro* Study on Canine Teeth

**DOI:** 10.1155/2016/8594087

**Published:** 2016-09-26

**Authors:** Maciej Janeczek, Piotr Kosior, Dagmara Piesiak-Pańczyszyn, Krzysztof Dudek, Aleksander Chrószcz, Agnieszka Czajczyńska-Waszkiewicz, Małgorzata Kowalczyk-Zając, Aleksandra Gabren-Syller, Karol Kirstein, Aleksandra Skalec, Ewelina Bryła, Maciej Dobrzyński

**Affiliations:** ^1^Department of Biostructure and Animal Physiology, Wroclaw University of Environmental and Life Sciences, Kożuchowska 1, 51-631 Wroclaw, Poland; ^2^Department of Conservative Dentistry and Pedodontics, Wroclaw Medical University, Krakowska 26, 50-425 Wroclaw, Poland; ^3^Faculty of Mechanical Engineering, Technical University of Wroclaw, Łukasiewicza 5, 50-371 Wroclaw, Poland; ^4^Dental Clinic Adentis, Surowieckiego 8, 02-785 Warszawa, Poland; ^5^Dental Practice, Bartąska 2/22, 10-699 Olsztyn, Poland; ^6^Private Dental Clinic Maciej Kozłowski, Spokojna 23, 56-400 Oleśnica, Poland

## Abstract

Measurements of the root canal during endodontic treatment have a significant influence on the course of the therapeutic process as well as on its final result in both human and veterinary medicine. The apical constriction should be the termination point for the preparation and filling of the root canal. This research was conducted with the use of a Septodont kit consisting of a small chamber filled with the examined solution in which a healthy second incisor was placed. The step back method was applied for the root canal preparation and master apical file of 30 was used. The working length was 22 mm. The examination was conducted with the use of steel as well as nickel titanium hand instruments. Different irrigation solutions and two types of apex locators were used. Measurements of the working length of the root canal showed dependence on the size of the instrument. Examinations carried out in various environments showed that analogical measurements were obtained only for sodium hypochlorite solutions. In other environments the measured sections were shortened. Comparative examinations with the use of steel instruments demonstrated insignificant measurement differences. Compared to these results, the measurements in nickel titanium group were characterized by more considerable deviations.

## 1. Introduction

Root canal treatment is a standard procedure applied in veterinary dentistry. The indication for its implementation includes pulp disease and dental injuries [1–4]. Working length measurement is significantly important for successful outcome of root canal treatment [5]. According to contemporary views, the place up to which the root canal preparation and obturation should be performed is the narrow apical part of the root canal called the apical constriction (AC) which is located 0.5–1.0 mm coronal to the apical foramen [6–9]. Some authors [10] even believe that root canal preparation and obturation up to the level of the apical foramen is also acceptable, if it does not cause irritation of periapical tissues or overfilling of the root canal with the obturation material. Insufficient working length results in residual (necrotic or vital) tissue in the root canal, improper root canal preparation and obturation, formation of periapical lesions, posttreatment pain, and the spread of infection. Nevertheless, an incorrect working length may cause injury to the periapical tissues, bleeding, pain, extended treatment period resulting in reinfection, and a possibility of canal overfilling and extrusion of endodontic material beyond the apical region [9–11]. Due to high species variability, it is difficult to determine a standard tooth root canal length in dogs. Therefore, verification of the root canal length plays a major role in root canal treatment in dogs [1, 5]. The estimation of the root canal working length is based on two methods: radiological and endometric methods. The first one uses radiographic images taken in different projections in order to determine the proper root canal length. This method is based on radiographic image with an endodontic file that locates the apical foramen and facilitates the analysis of anatomic structures and systems. The disadvantages of this method are lack of possibility to reflect the actual length of the root, difficulty with setting the proper projection, two-dimensional image, necessity for exposure to radiation, and occurrence of interpretational differences [9–12]. The endometric method assumes determining the working length of the root canal with the use of electric devices, the so-called endometers (*electronic apex locators* (*EALs*)). The first devices were introduced in the 1960s. The resistance of electric current passing between a passive electrode (a hook placed on the patient's lip) and an active electrode (a clip or holder placed on the endodontic instrument inserted into the root canal) was used for performing the measurement. At the moment of reaching the apical constriction, the resistance fell to 6.5 Ω, which was indicated by the device [13]. Unfortunately, high sensitivity to fluid generated incorrect measurements and made its common use difficult [14]. After implementing some modifications in the 1990s, third-generation endometers were introduced. These are frequency endometers based on measurements performed through a comparison of the values of two or more impedance measurements [7, 14, 15]. They are characterized by very low sensitivity to fluid, they allow recording measurements in the presence of incompletely removed pulp, and they enable multiple repetitions of the examination without any negative impact on the patient's health. Moreover, they do not locate the apical foramen but the apical constriction which is the proper termination point for the root canal preparation and filling. The accuracy of root canal length measurement is set on the level of 80–100% [8, 14, 15]. However, it is worth bearing in mind that, in some clinical situations, the use of a frequency apex locator increases the probability of a measurement error. It concerns root canals with root apex obliteration, in immature permanent teeth with open apices, teeth with apical resorption [10, 15].

The authors conducted an examination aimed at assessing the influence of the size and type of instruments and the type of irrigation solution on the accuracy of electrical measurement of the working length.

## 2. Materials and Methods

**Figure 1:**
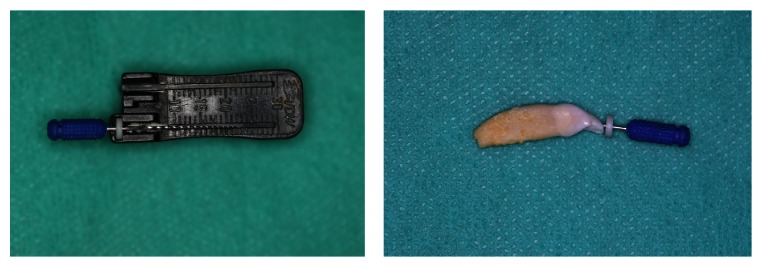
Working length of the instrument (master apical file (MAF)) was 22 mm and it was established with the use of VDW endodontic ruler.

**Figure 2:**
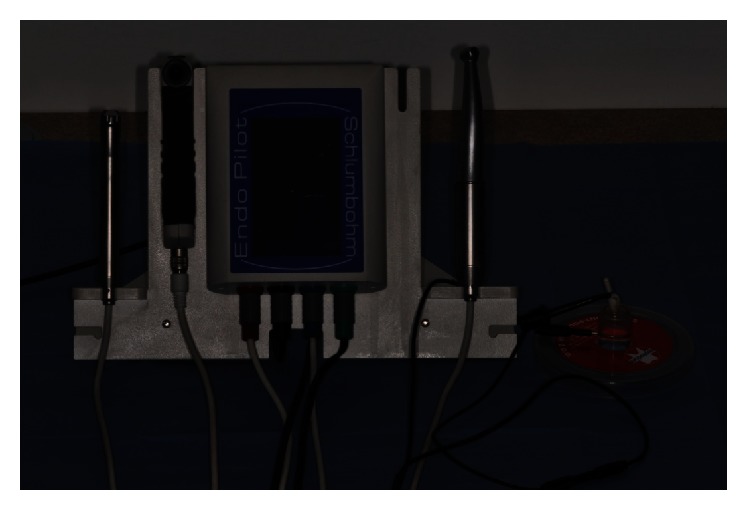
Septodont measuring kit consisting of a chamber filled with a selected solution, a passive electrode connected with the chamber, and an active electrode joined with the endodontic instrument placed in a root canal of the tooth and connected with the Endopilot apex locator (Schlumbohm, Brokstedt, Germany).

**Figure 3:**
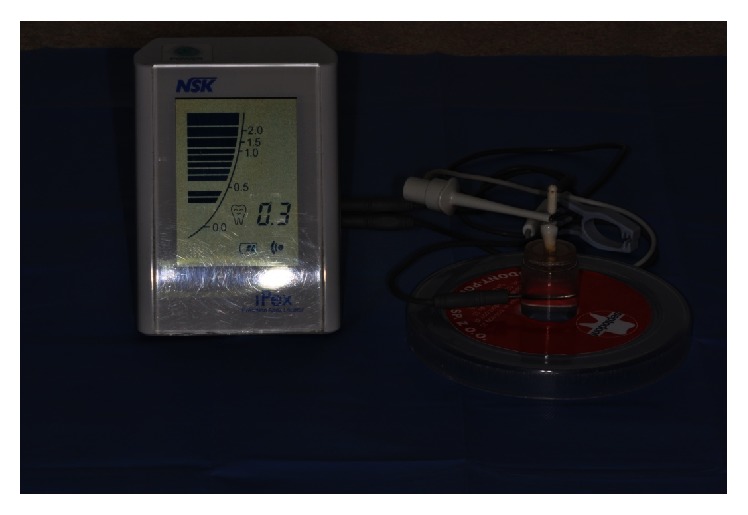
Septodont measuring kit consisting of a chamber filled with a selected solution, a passive electrode connected with the chamber, and an active electrode joined with the endodontic instrument placed in a root canal of the tooth and connected with the iPex apex locator (NSK, Tochigi, Japan).

The examination was carried out with the use of a Septodont experimental kit consisting of a chamber filled with the examined solution and a healthy upper second incisor of a dog placed in it. The step back method was applied to prepare the root canal from the size of 08 to the size of 40 with the taper of 02, obtaining MAF = 30 (master apical file), that is, the last root canal preparation instrument along the whole working length. The working length was 22 mm and it was determined by the use of VDW endodontic ruler (Figure 1). The passive electrode was connected to the chamber, which was filled with the selected solution, and the active one was connected to an endodontic file placed in the root canal of the tooth. Two types of apex locators were used in the experiment: Endopilot® (Schlumbohm, Brokstedt, Germany) and iPex® (NSK, Tochigi, Japan) (Figures 2 and 3). Every measurement was taken three times, and average values were recorded in Tables 1 [Table tab2] [Table tab3]–4. The following instruments were used in the examination: steel S-files (Poldent®), steel H-files (Mani®), steel K-files (Medin®), steel K-reamer (Mani), and NiTi H-files (Polintech®). The following irrigation solutions were used: 2.0% sodium hypochlorite (Chema-Elektromet), 5.25% sodium hypochlorite (Cerkamed), 40.0% citric acid (Cerkamed), 0.2% chlorhexidine digluconate—Gluco-Hex (Cerkamed), 0.9% NaCl (Fresenius Kabi), 3.0% hydrogen peroxide (Avena), 15.0% disodium edentate (Chema-Elektromet), and 70.0% isopropyl alcohol—2-propanol C_3_H_7_OH (Cerkamed).

The STATISTICA (StatSoft, Inc., Tulsa, USA) software was used for statistical analysis. The normality of empirical distributions was verified with the use of the Shapiro-Wilk test. The significance of differences between average values in two groups was verified with Student's* t*-test, and in case of a bigger amount of groups, the analysis of variance (ANOVA) was implemented. Pearson correlation coefficients were calculated for determining the magnitude and direction of the correlation of two variables. *p* values below 0.05 were considered significant.

## 3. Results and Discussion

**Figure 4:**
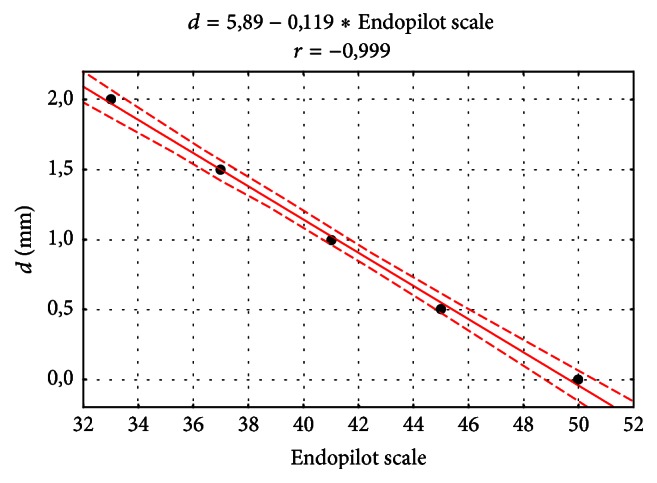
Correlation diagram of the distance between the instrument and the apical constriction (mm) with the indication of the Endopilot apex locator and the regression equation.

The values reflecting the distance between the file tip and the apical constriction obtained when taking measurements with the use of the Endopilot apex locator are presented on a point scale. The change in the readings of the device is proportional to the working length of the instrument on average; it corresponded to 4-5 units of the scale per each 0.5 mm calculated from 2 mm coronal to the apical constriction. The indications of the Endopilot apex locator were converted into the distance* d *(mm) between the instrument and the apical constriction and the value of the Pearson correlation coefficient *r* with the use of a correlation diagram (Figure 4) which contains a regression equation. The *r* coefficient is close to 1, which means that there is a strong linear correlation between both parameters (*p* < 0.0001).

The measurements of the working length of the prepared root canal performed with the use of a steel S-file (Poldent) in the 5.25% NaOCl environment with two types of EALs demonstrated a dependence on the size of the instrument. The most accurate result was obtained for the MAF (with the size of 30/22 mm) for which the distance from the apical constriction was 0.5 mm for both iPex and Endopilot (actual length and electrical length values were equal). The difference in the values of the measurements taken with the use of an instrument with the smallest (08) and largest (30) diameter for the working length of 22 mm was 1.0 mm for Endopilot and 0.9 mm for iPex. The greatest error was recorded for the measurements performed with the instruments with the smallest diameter in comparison to the size of the prepared root canal.  Table 1 shows the average ± standard deviation of the measurement results of the distance between the instrument and the apical constriction obtained in the 5.25% NaOCl environment with the use of steel S-files (Poldent) with the size ranging from 08 to 30 in accordance with ISO and with the use of two apex locators, Endopilot and iPex NSK, as well as the results of comparison made with the use of Student's* t-*test for dependent variables.

The measurements of the working length were performed in various environments in order to determine the influence of irrigation solutions used in endodontic treatment on the accuracy and stability of the results. The comparison of the readings from Endopilot and iPex NSK demonstrated that analogical measurements were obtained only in the 2.0% and 5.25% NaOCl environment. When using the steel file with the size of 30/22 mm as an active electrode, the distance between the apical constrictions for the 2.0% and the 5.25% NaOCl solution was 0.5 mm. In the 40.0% citric acid, 0.2% chlorhexidine, 3.0% hydrogen peroxide, 15.0% disodium edentate, and 0.9% NaCl groups, electrical conductivity was changed, which resulted in increasing the distance between file tip and apex, and it was, respectively, 1.5 mm, 2.0 mm, 2.0 mm, 1.5 mm, and 1.2 mm for iPex and above 2.0 mm for Endopilot in all cases apart from disodium edentate. For Endosal, the obtained measurement was 1.4 mm coronal to the apex. In the environment of isopropyl alcohol, the readings on both devices showed zero, independently of the distance between the instrument and the apical constriction of the examined tooth. The average ± standard deviation of the distance between the file tip and the apical constriction measured with the use of a steel file with the size of 30/22 mm for different irrigation solutions and the results of comparison made with Student's* t*-test for dependent variables are shown in Table 2.

**Figure 5:**
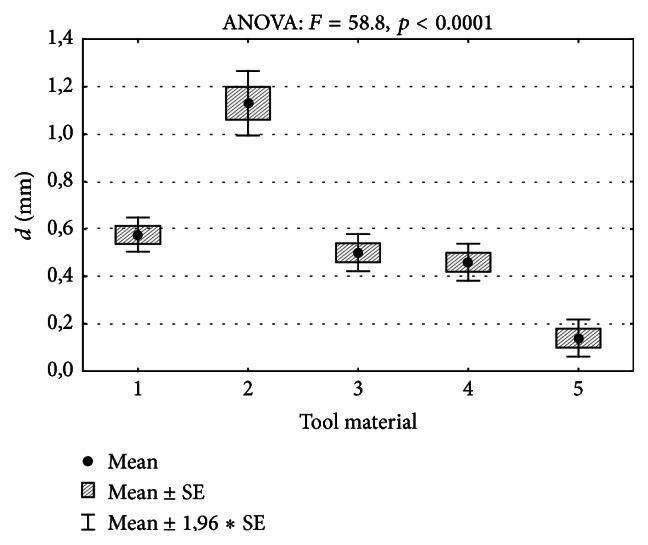
Comparison of measurement results of the distance from the apical constriction* d* (mm) obtained with the use of the Endopilot apex locator and the results of the analysis of variance, where 1 stands for H-file Mani steel, 2 stands for H-file Polintech NiTi Flexible, 3 represents K-file Medin steel, 4 represents K-reamer Mani steel, and 5 represents S-file Poldent steel.

In the course of examination, comparative measurements with the use of steel files and reamers, as well as nickel titanium instruments, were also taken in order to determine the influence of the type of active electrode on the obtained result. The measurement was performed in the 5.25% NaOCl environment with the use of the Endopilot apex locator. Insignificant measurement differences were observed in the group of steel instruments of various shapes of the working part and they remained at the level of 0.2–0.3 mm (comparing to MAF length). A slightly higher deviation was observed in the case of NiTi instruments (1.1 mm from the apex, i.e., shorter than the length of the master apical file by 0.6 mm) (Table 3).  Figure 5 shows the comparison of the obtained measurement results and the results of the analysis of variance.  Table 4 shows the results of multiple comparisons conducted with the use of a* post hoc* test.

The results of the measurements performed with the use of the H-file Polintech NiTi Flexible (2) are significantly higher than the rest (*p* < 0.001). The results obtained with the S-file Poldent steel (5) instrument, in turn, are lower than the rest. The differences between the measurement results obtained with instruments made of materials (1), (3), and (4) do not significantly differ (*p* > 0.05).

The accuracy of the measurements performed with the use of electrical methods depends on several factors including the type and size of the root canal instrument used for taking the measurement, the diameter of the apical foramen, the shape of the root canal, and the type of irrigation solution [11, 12, 16]. Some authors [8, 14, 17–19] report that the size of the apical foramen and the endodontic instrument determine the obtained result. With regard to the size of the apical foramen, there are still discussions about the borderline value which enables and determines the proper examination. According to some authors [17, 20, 21], the apical foramen with the diameter not exceeding 0.2 mm does not have an impact on the measurements. It is only a size of more than 0.2 mm that will cause an increase in the distance measured between the file tip and the apical foramen. In contrast to this theory, Herrera et al. [18, 19] did not observe a significant influence of the size of the apical opening which was above 0.2 mm on the sensitivity of measurement of the working length. The differences obtained by particular researchers may, according to Kolanu et al. [14], not be caused by the size of the foramen itself, but also by the surface of contact of the active electrode with the walls. Enlargement of the apical foramen diameter leads to collaterality of the canal walls and thus contributes to the measurement error [22]. In the conducted experiments, it was concluded that the increase in the instrument diameter at the same working length corresponds to a shorter distance from the apex indicated by a given apex locator. Such dependencies were observed in the case of both apex locators. A reading which was equal to the actual one was obtained in the case of an instrument with the size of 30 in accordance with ISO, which corresponds to MAF (Table 2). Similar results were obtained by McDonald [23] and Ebrahim et al. [8] pointed out that the diameter of the root canal increases and causes the length decreases measured with smaller size files. These authors recommended the use of files with sizes comparable with the root canal diameter, claiming that this would result in more accurate readings. They concluded that the measurements of the location of the apical constriction performed with an instrument which was smaller than the diameter of the prepared canal lead to errors, shortening of the distance. Using endodontic instruments whose size is adjusted, that is, that close to the root canal diameter, enables one to perform accurate measurements even in the presence of fluids, for example, blood. Ebrahim et al. [8] obtained a major measurement error when using small files (K-file 10) as active electrodes in root canals that were ultimately prepared to the size of 80. It was 0.19 mm for groups of teeth irrigated with NaOCl and 1.11 mm for teeth with human blood, respectively. Using MAF to perform the measurements reduced the error below 0.03 mm in both groups.

The type of instrument and the material it is made of may also have an influence on the accuracy of the measurements. The instruments used in endodontic treatment are made of various materials, including high quality stainless steel, carbon steel, chromium and nickel alloys, and nickel titanium alloys. Nickel titanium is an alloy consisting of 54% nickel and 46% titanium which is characterized by shape memory, high flexibility, and resistance to fracture. Dental stainless steel is an alloy that contains 73% iron, 9% nickel, and 18% chromium. Both materials are characterized by endurance and resistance to biofluids of the oral cavity as well as chemical and physical factors occurring in the process of disinfection and sterilization. Nickel titanium instruments additionally show increased flexibility; they are harder and more resistant; they have better cutting ability and the so-called shape memory (they always return to the initial shape). Moreover, they are more economical, as they may be utilized 2-3 times longer. On the other side, steel instruments are resistant and they are characterized by very good cutting abilities. Their flexibility depends mainly on the size; however, they may be adjusted to the shape of the canal within safety limits [10]. Our research has shown insignificant differences for steel instruments (of different manufacturers) when comparing the indications of the Endopilot apex locator for various endodontic instruments with the same working length (22 mm) that corresponded to MAF in the 5.25% NaOCl solution. Their length was* ca*. 0.2–0.3 mm shorter than earlier estimated MAF length. In comparison to the steel instruments, the measurements performed with nickel titanium ones were 0.5–0.8 mm shorter, which indicated shortening of the working length by approximately 1 mm. The research conducted by Nekoofar et al. [24] demonstrated that in 94.4% cases usage of nickel titanium instruments caused deviation of the measurement by ±0.5 mm from the determined working length, and in the case of steel instruments, the same applied to 91.6% cases.

Due to the antibacterial and lubricating features, as well as the ability to dissolve vital tissue, using a wide range of irrigation solutions in endodontic treatment significantly facilitates proper preparation of the root canal. However, the presence of any fluids may hinder the use of apex locators and obtaining accurate measurements. The opinions of researchers regarding this issue are mixed. Some authors [7, 8, 25, 26] believe that the least significant impact is achieved when using the NaOCl solution regardless of its concentration. It comes from the fact that it is a solution characterized by high electrical conductivity and with the potential to penetrate into dentinal tubules and decrease electrical impedance of the root canal walls as well as generate better electrical contact with periapical tissues [8, 16, 27]. Khattak et al. [28] and Khursheed et al. [29] obtained the best results in the 0.2% chlorhexidine environment. In the environment of a 3.0% solution of NaOCl, the difference between the measured and the actual length was significantly larger. In our research, the most actual and accurate measurement of the working length was obtained in both sodium hypochlorite solutions, that is, 2.0% and 5.25% for Endopilot and iPex. In other examined solutions, the compatibility was lower. For 15.0% disodium edentate it was 1.4 mm and 1.5 mm coronal to the apical constriction, and for 0.2% chlorhexidine and 3.0% hydrogen peroxide, it was 2.0 mm and above 2.0 mm, respectively. Indications which were significantly different from the actual ones were obtained in the 40.0% citric acid solution and saline. In the environment of isopropyl alcohol, the readings on both devices were zero, independently of the distance of the instrument from the apex of the examined tooth. It means that isopropyl alcohol is a complete insulator.

## 4. Conclusions

The new generation apex locators enable dentists to perform accurate measurements even in moist root canals or in the presence of residual nonremoved pulp. The study carried out by the authors demonstrated the highest stability of measurements in the NaOCl environment, in the case of both 2.0% and 5.25%, which seems to be the most optimum one. It was also concluded that the use of steel instruments whose size corresponds to MAF (master apical file) brings the best results. The awareness of these factors may significantly facilitate root canal treatment, prevent complications, and help obtain satisfactory results, in both human and veterinary medicine.

## Figures and Tables

**Table 1:** Basic statistics of the measurement results of the distance between the instrument and the apical constriction performed in the 5.25% NaOCl environment with the use of steel S-files (Poldent) with the size ranging from 08 to 30 in accordance with ISO and with the use of two apex locators: Endopilot and iPex NSK as well as the results of comparison made with the use of Student's *t*-test for dependent variables.

Instrument size	Endopilot	iPex NSK	*t*	*p*
*l* = 20 mm, ⌀8 mm	3.23 ± 0.03	2.58 ± 0.03	**27.577**	**<0.001**
*l* = 20 mm, ⌀10 mm	2.93 ± 0.12	2.22 ± 0.10	**7.985**	**0.001**
*l* = 20 mm, ⌀15 mm	3.02 ± 0.12	2.07 ± 0.15	**8.593**	**0.001**
*l* = 20 mm, ⌀20 mm	2.58 ± 0.10	2.00 ± 0.05	**8.750**	**0.001**
*l* = 20 mm, ⌀25 mm	2.48 ± 0.06	1.98 ± 0.03	**13.416**	**<0.001**
*l* = 20 mm, ⌀30 mm	2.08 ± 0.06	1.88 ± 0.13	2.370	0.077
*l* = 21 mm, ⌀8 mm	1.82 ± 0.08	1.70 ± 0.10	1.606	0.184
*l* = 21 mm, ⌀10 mm	1.73 ± 0.15	1.67 ± 0.03	0.499	0.499
*l* = 21 mm, ⌀15 mm	1.52 ± 0.06	1.38 ± 0.03	**3.578**	**0.023**
*l* = 21 mm, ⌀20 mm	1.37 ± 0.03	1.30 ± 0.05	2.000	0.116
*l* = 21 mm, ⌀25 mm	1.25 ± 0.00	1.23 ± 0.03	1.000	0.374
*l* = 21 mm, ⌀30 mm	1.12 ± 0.08	1.22 ± 0.03	−2.121	0.101
*l* = 22 mm, ⌀8 mm	1.48 ± 0.08	1.38 ± 0.03	2.121	0.101
*l* = 22 mm, ⌀10 mm	1.38 ± 0.08	1.32 ± 0.03	1.414	0.230
*l* = 22 mm, ⌀15 mm	1.15 ± 0.05	1.03 ± 0.03	**3.500**	**0.025**
*l* = 22 mm, ⌀20 mm	0.93 ± 0.03	0.83 ± 0.03	**4.243**	**0.013**
*l* = 22 mm, ⌀25 mm	0.78 ± 0.03	0.62 ± 0.03	**7.071**	**0.002**
*l* = 22 mm, ⌀30 mm	0.53 ± 0.03	0.52 ± 0.03	0.707	0.519

**Table 2 tab2:** Basic statistics of the distance between the endodontic device and the apical constriction measured with the use of a steel file with the size of 30/22 mm for different irrigation solutions.

Irrigation solution	Endopilot	iPex NSK	*t*	*p*
2.0% NaOCl	0.51 ± 0.02	0.49 ± 0.02	1.604	0.184
5.25% NaOCl	0.50 ± 0.05	0.52 ± 0.01	−0.611	0.574
40.0% citric acid	2.10 ± 0.04	1.51 ± 0.08	**11.693**	**<0.001**
2.0% Gluco-chex	2.11 ± 0.05	1.99 ± 0.06	2.668	0.056
Isopropyl alcohol	0	0	—	—
3.0% H_2_O_2_	2.13 ± 0.03	2.02 ± 0.08	2.220	0.091
15.0% Endosal	1.37 ± 0.10	1.49 ± 0.06	−1.886	0.132
0.9% NaCl	2.07 ± 0.03	1.23 ± 0.05	**24.362**	**<0.001**

Statistically significant differences between the measurement results obtained with the use of the Endopilot and iPex NSK apex locators were observed in the 40.0% citric acid and 0.9% NaCl solutions (*p* < 0.001).

**Table 3 tab3:** Measurements of the distance from the apical constriction performed with the use of steel and NiTi instruments with the size of 30/22 mm and with the use of Endopilot apex locator in the 5.25% NaOCl environment.

	Material					ANOVA
	(1) H-file Mani steel	(2) H-file Polintech NiTi Flexible	(3) K-file Medin steel	(4) K-reamer Mani steel	(5) S-file Poldent steel	
Apex locator reading	44.7 ± 0.5	40.0 ± 0.8	45 ± 1	46 ± 1	48 ± 1	*F* = 58.8
Distance *d* (mm)	0.58 ± 0.06	1.13 ± 0.12	0.50 ± 0.07	0.46 ± 0.07	0.14 ± 0.07	*p* < 0.001

**Table 4:** Results of multiple comparisons with the use of a *post hoc* test.

Material		(1) *M* = 0.58	(2) *M* = 1.13	(3) *M* = 0.50	(4) *M* = 0.46	(5) *M* = 0.14
1	(1)		**0.000**	0.773	0.442	**0.001**
2	(2)	**0.000**		**0.000**	**0.000**	**0.000**
3	(3)	0.773	**0.000**		0.971	**0.002**
4	(4)	0.442	**0.000**	0.971		**0.005**
5	(5)	**0.001**	**0.000**	**0.002**	**0.005**	

Tukey HSD test. Variable: *d* (mm).

Bold differences are significant at *p* < 0.05.
